# Viral Protein Inhibits RISC Activity by Argonaute Binding through Conserved WG/GW Motifs

**DOI:** 10.1371/journal.ppat.1000996

**Published:** 2010-07-15

**Authors:** Ana Giner, Lóránt Lakatos, Meritxell García-Chapa, Juan José López-Moya, József Burgyán

**Affiliations:** 1 Centre for Research in Agricultural Genomics, CRAG, CSIC-IRTA-UAB, Barcelona, Spain; 2 Agricultural Biotechnology Centre, Gödöllő, Hungary; 3 Instituto di Virologia Vegetale, Torino, Italy; University of Kentucky, United States of America

## Abstract

RNA silencing is an evolutionarily conserved sequence-specific gene-inactivation system that also functions as an antiviral mechanism in higher plants and insects. To overcome antiviral RNA silencing, viruses express silencing-suppressor proteins. These viral proteins can target one or more key points in the silencing machinery. Here we show that in *Sweet potato mild mottle virus* (SPMMV, type member of the *Ipomovirus* genus, family *Potyviridae*), the role of silencing suppressor is played by the P1 protein (the largest serine protease among all known potyvirids) despite the presence in its genome of an HC-Pro protein, which, in potyviruses, acts as the suppressor. Using in vivo studies we have demonstrated that SPMMV P1 inhibits si/miRNA-programmed RISC activity. Inhibition of RISC activity occurs by binding P1 to mature high molecular weight RISC, as we have shown by immunoprecipitation. Our results revealed that P1 targets Argonaute1 (AGO1), the catalytic unit of RISC, and that suppressor/binding activities are localized at the N-terminal half of P1. In this region three WG/GW motifs were found resembling the AGO-binding linear peptide motif conserved in metazoans and plants. Site-directed mutagenesis proved that these three motifs are absolutely required for both binding and suppression of AGO1 function. In contrast to other viral silencing suppressors analyzed so far P1 inhibits both existing and *de novo* formed AGO1 containing RISC complexes. Thus P1 represents a novel RNA silencing suppressor mechanism. The discovery of the molecular bases of P1 mediated silencing suppression may help to get better insight into the function and assembly of the poorly explored multiprotein containing RISC.

## Author Summary

RNA silencing is an evolutionarily conserved sequence-specific gene-inactivation system that also functions as a major antiviral mechanism in higher plants and insects. Viral RNAs are processed by Dicer-like proteins into small interfering (si) RNAs, which trigger the RNA-induced silencing complex (RISC) assembly. Then siRNA loaded RISC inactivates cognate viral RNA. However, viral silencing suppressors evolved to counteract with RNA silencing targeting one or more key points in the silencing machinery. Here we show that in *Sweet potato mild mottle virus*, the role of silencing suppressor is played by P1 protein and it works by inhibiting si/miRNA-loaded RISC through targeting Argonaute 1 (AGO1). We confirmed using immunoprecipitation and *in vitro* binding assays that the interaction between P1 and small RNA loaded AGO1 is specific and direct. The suppression activity mapped to the N-terminal part of P1 containing three WG/GW motifs that resemble the AGO-binding linear peptide motif conserved in metazoans and plants. Site-directed mutagenesis proved that these three motifs are essential for both binding and suppression of AGO1 function. P1 protein is the only silencing suppressor identified so far that inhibits active RISC and this is the first demonstration of a WG/GW protein having negative effect on RNA silencing.

## Introduction

Most eukaryotes, including plants, make use of a well-conserved RNA silencing mechanism to regulate many essential biological processes, ranging from development and control of physiological activities, to responses to abiotic and biotic stress, in particular antiviral defense [1], [2].

Antiviral defense in plants begins with the activity of RNase III type Dicer-Like (DCL) enzymes, which target viral RNAs [3], [4]. Concerted action of the DCL4, DCL2, DCL3 and occasionally DCL1 enzymes results in the appearance of 21–24 nt small interfering RNAs (siRNAs), the central components of the RNA silencing pathway [4], [5]. These viral siRNAs subsequently loaded to endogenous AGO proteins, which are catalytic component of RNA-induced silencing complex (RISC) [6], [7]. AGO1 and AGO7 are suggested to be involved in antiviral silencing [8], [9], [10] although previous study failed to detect viral siRNAs in tagged AtAGO1 [11]. It has been also shown that AGO7 favors less structured RNA targets, while AGO1 is capable of targeting viral RNAs with more compact structures [9]. AGO proteins are responsible for targeting RISC to viral genomes (either RNA or DNA), and exert their action either through cleavage or inhibition of translation [12]. The RNA-dependent RNA polymerases (RDRs) of the host also play important roles in antiviral RNA silencing, being involved in production of secondary viral siRNA [13], [14], [15], [16], [17], [18].

Viruses have evolved suppressors to counteract the RNA-silencing defense of the host [1], [2], [19]. The more than 35 viral silencing-suppressor families so far identified use different strategies to inhibit RNA silencing [2], [20]. Sequestering siRNAs by siRNA-binding suppressors is a very common way to inhibit RISC assembly [21], [22], but other mechanisms have been described, such as inhibiting the biogenesis of 21 nt siRNA species [4], [20], [23]. Other suppressors inhibit RNA silencing through protein-protein interaction. The 2b protein of CMV strain Fny is suggested to inhibit RISC activity via physical interaction with the PAZ domain of the plant AGO1 protein [10]. *Polerovirus* P0 suppressor protein has been suggested to target PAZ domain of AGO1 and directing its degradation [24], [25].

**Figure 1:**
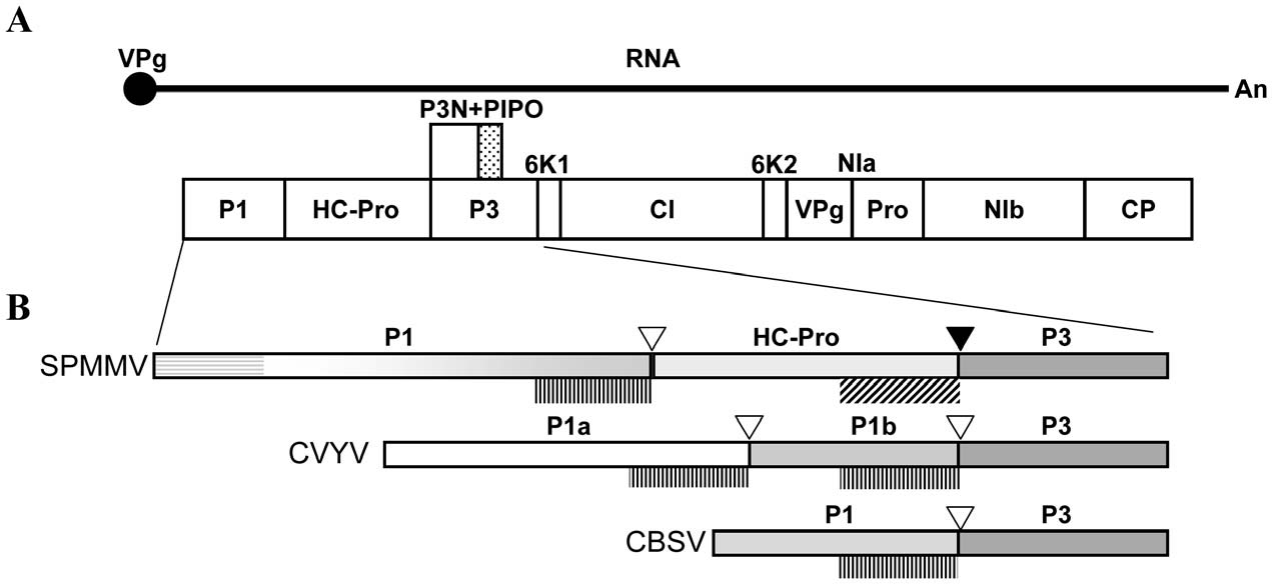
Genome structure of SPMMV ipomovirus and sequence peculiarities of the N-terminal part of its polyprotein which includes the P1 protein. (A) Graphical representation of the genome organization of viruses of the family *Potyviridae*. The ssRNA genome of a generic potyvirid is depicted as a solid line with a 5′ covalently linked VPg and a 3′ polyA tail. The viral polyprotein is shown as a large box divided in the different mature protein products, and the small dotted box above represents the alternative P3-embeded ORF known as PIPO, expressed after a frameshift. (B) Details of genomic variants found in the N-terminal part of ipomoviruses. Examples of individual viruses (acronyms at the left) of each type of genomic organization are shown, and the relative sizes of proteins are represented proportionally. The proteinase domains and cleavage sites are indicated respectively by plotted vertical lines (below) and empty triangles (above) in the case of P1-like serine proteinases, or by oblique lines (below) and solid triangles (above) for the HC-Pro-like cysteine proteinase in SPMMV. Pattern consistency is used to represent homologies, and the gradational scale in the C-terminal part of SPMMV P1 indicates that it presents partial homology in this region to the P1b of CVYV (and also to the P1b of SqVYV, not shown), and to the P1 of CBSV, while the horizontal lines represents a characteristic N-terminal extension with partial homology to the N-terminal part of the P1 protein of the potyvirus SPFMV.

The *Potyviridae* is the largest family of plant RNA viruses; in most members, the single-stranded RNA genome is about 10 kb in size and encodes a single polyprotein that is processed into at least 9 mature proteins [26] (Figure 1). In the genus *Potyvirus*, the multifunctional HC-Pro (helper component-proteinase) was the first viral product to be recognized as a silencing suppressor [27], [28], [29]. The genome of *Cucumber vein yellowing virus* (CVYV), genus *Ipomovirus*, family *Potyviridae*, lacks HC-Pro but contains two P1-type proteases [30], properties shared by at least one other ipomovirus [31]. In CVYV, the second P1 cistron (P1b) was found to suppress RNA silencing [30] with a mode of action resembling that of the HC-Pro of potyviruses [32]. Interestingly, the type member of the genus *Ipomovirus*, *Sweet potato mild mottle virus* (SPMMV), possesses an HC-Pro region and a single large P1 serine protease [33].

The peculiarities of the SPMMV genome that incorporates the largest P1 region among all known members of the family together with a typical HC-Pro region (Figure 1), prompted us to study how this virus might deal with the RNA-silencing machinery in its hosts.

In the present study, we show that the large P1 protein of SPMMV possesses silencing-suppressor activity, while its HC-Pro protein does not, on its own. Using various reporter systems, we show that *in vivo* P1 inhibits target RNA cleavage mediated by RISC complexes loaded with either endogenous miRNA or with virus-derived siRNA. Moreover, suppression activity mapped to the N-terminal half of P1, a region containing three WG/GW motifs that mimics AGO-binding linear peptide motif conserved both in metazoans and plants [34], [35]. We have also determined that the WG/GW motifs at the very N-terminal end in P1 are required for AGO1 binding and for silencing-suppression, suggesting that P1 may use the conserved WG/GW motif binding surface of Ago proteins to inhibit RISC activity.

## Results

A partial sequence of 3633 nucleotides was obtained from a plant infected with SPMMV African isolate 130 after RT-PCR amplification with primers designed to flank the first two cistrons of the viral polyprotein. The N-terminal part of this sequence shares structure with the only SPMMV whole genome sequence available in databanks [33]. It presents a large P1 cistron encoding 743 amino acids (15 residues more than the published sequence, starting at position 362), followed by a 453 amino acid HC-Pro cistron, which is more similar in size to other potyviral HC-Pros. The expected cleavage sites and the corresponding residues for the active sites of P1 and HC-Pro proteases [36] could be recognized in the sequence. However, SPMMV HC-Pro lacks the conserved FRNK box characteristic of potyviral HC-Pros and required for small RNA binding and symptom development [37]. These characteristics make SPMMV unique among the *Potyviridae*, including other ipomoviruses (Figure 1). Sequences are compared in Figure S1.

### P1 is the silencing suppressor of SPMMV

**Figure 2:**
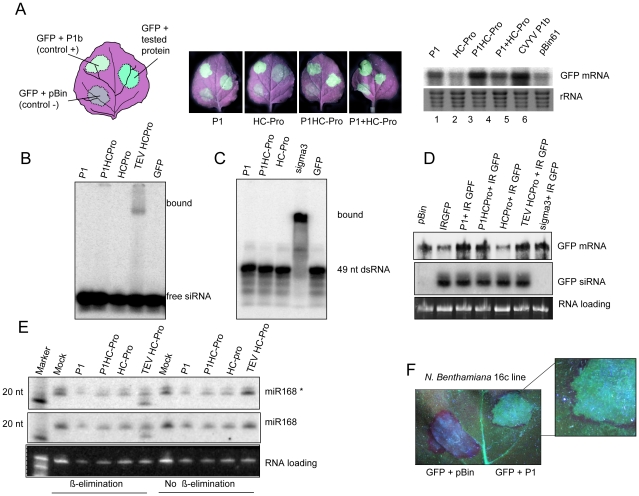
SPMMV P1 suppresses RNA silencing by mechanisms that diverge from other viral suppressors. (A) P1 and HC-Pro proteins of SPMMV were agroinfiltrated into *N.benthamiana* leaves (according to the schematic drawing shown at the left) along with a reporter 35S-GFP construct to visualize (central panels) and to analyze by northern blot (right panel) their effects on RNA silencing. The P1b suppressor of CVYV was used as positive control, while an empty pBin61 served as negative control. (B) ^32^P-labeled ds 21nt siRNA was incubated with extracts of leaves infiltrated with constructs expressing SPMMV P1, SPMMV HC-Pro, both P1 and HC-Pro of SPMMV in cis (P1HC-Pro), TEV HC-Pro and GFP. Bound complexes were electrophoretically separated on a native gel. (C) ^32^P-labeled ds 49 nt RNA was incubated with extracts of leaves infiltrated with P1, P1HC-Pro, and HC-Pro of SPMMV, Reovirus sigma3 and GFP. Bound complexes were electrophoretically separated on a native gel. (D) RNA preparations from leaves of 16c transgenic lane of *N. benthamiana* plants expressing GFP infiltrated with SPMMV P1, SPMMV HC-Pro and TEV HC-Pro were analyzed by northern blot with GFP-specific probes for the presence of mRNA and siRNA (upper and central panels); loading charge is shown at the lower panel. (E) Analysis of 3′ methylation of miR168 in the presence of the indicated viral suppressors. RNA samples with or without oxidation and ß-elimination were separated in 12% polyacryamide gel and then blotted. Small RNA blot was hybridized with LNA oligonucleotide probes detecting miR168 star (miR168*) and miR168 mature strands. Faster miRNA bands indicate the non-methylated miRNAs. (F) Detail of a leaf from a *N. benthamiana* 16c plant under UV light at 8 days after co-agroinfiltration with the reporter 35S-GFP and pBin empty vector (left) or SPMMV P1 (right). The development of the red halo around the agroinfiltrated patch indicated that SPMMV P1 did not abolish movement of the signal.

To investigate whether P1 and/or HC-Pro serve (s) as RNA silencing suppressor for SPMMV, we used the standard Agrobacterium coinfiltration assay [22]. The complete cistrons for P1 and HC-Pro were cloned into binary vectors and the resulting expression constructs were transferred into *A. tumefaciens*. Cultures of *A. tumefaciens* able to express GFP from a 35S-promoter GFP binary plasmid were mixed with cultures transformed with our SPMMV constructs before infiltration into *Nicotiana benthamiana* leaves. In this assay both fluorescence and RNA analysis identified SPMMV P1, but not SPMMV HC-Pro, as the suppressor of RNA silencing (Figure 2A). Weak fluorescence and low GFP mRNA levels were observed in patches infiltrated with the pBin61 empty vector (negative control), and strong suppressor activity and increased GFP mRNA level were detected in patches infiltrated with a construct expressing the P1b of CVYV (positive control) [32].

### SPMMV P1 differs in mode of action from RNA-silencing suppressors of related viruses

Experiments designed to compare in parallel the suppression activity of SPMMV P1 with that of a suppressor from a potyvirus, the HC-Pro protein of *Tobacco etch virus* (TEV) were performed next. First, we checked *in vitro* if SPMMV proteins could bind either typical 21 nt ds siRNAs, or longer dsRNAs. Extracts of *N. benthamiana* leaves infiltrated with Agrobacterium strains expressing different suppressors were tested for siRNA binding with labeled 21 nt ds siRNA, and the complexes were resolved on a native gel. As expected, TEV HC-Pro bound ds siRNA, while SPMMV P1 and HC-Pro did not show any siRNA binding activity (Figure 2B). The same extracts were then incubated with a labeled 49 nt dsRNA, and the putative complexes were analyzed on a native gel. In this case, formation of the expected RNA-protein complex only occurred between the 49 nt dsRNA and the Sigma3 protein of a Reovirus [38] used as positive control, but no complexes were detected in any of the other samples from constructs of P1 and HC-Pro of SPMMV (Figure 2C).

Next, we tested if P1 inhibits small RNA processing in leaves of transgenic *N. benthamiana* line 16C, expressing a GFP transgene, that were coinfiltrated with an Agrobacterium strain harboring a GFP inverted repeat (GFP-IR) construct. To this end, patches infiltrated with constructs expressing SPMMV P1, SPMMV HC-Pro (individual proteins), or SPMMV P1HC-Pro (a construct containing both proteins in cis), or with TEV HC-Pro, were analyzed after 3 days for the presence of GFP mRNA and siRNAs by Northern blotting. No reductions in siRNA processing from the GFP-IR were observed in all expressed proteins (Figure 2D), in contrast to the complete abolition observed in the positive control, which was the dsRNA-binding Sigma3 protein of Reovirus [38]. The 16c plants agroinfiltrated were also observed under UV light at 8 days after agroinfiltration to monitor the spreading of silencing signal. Importantly we found that the presence of SPMMV P1 did not abolish movement of the signal, and therefore silencing of the transgene around the agroinfiltrated area was observed (Figure 2F).

We also checked the capacity of the different viral proteins to inhibit *in vivo* 3′ modifications of small RNAs by the HEN1 methyltransferase [39], [40]. We expressed P1 along with different silencing suppressor proteins, and the 3′ end methylation status of the mature and star strands of miR168 were then evaluated by oxidation and beta elimination followed by Northern blotting of total RNA samples. Consistently with our previous results [41], TEV HC-Pro inhibited the 3′ methylation of both strands of the endogenous miR168. However , SPMMV P1 and HC-Pro had no effect on HEN1 mediated 3′ modification. (Figure 2 E).

### SPMMV P1 inhibits miRNA and siRNA programmed RISC activity

Our findings showed that in contrast to several other silencing suppressors SPMMV P1 does not interfere with the initial steps of the silencing pathway. Thus we hypothesized that it might compromise assembled RISC activity. Active RISC complexes are known to contain ss siRNA and are licensed to cleave the target RNA in a sequence specific manner [42]. Recently, we developed assays based on the transient expression of sensor constructs to test the effect of RNA-silencing suppressors on miRNA and siRNA loaded active RISC. Using these assays we have previously demonstrated that silencing suppressors with ds siRNA binding capacity such as the HC-Pro of potyviruses does not have any effect on miRNA and siRNA loaded RISCs *in planta* [22], [43].

**Figure 3:**
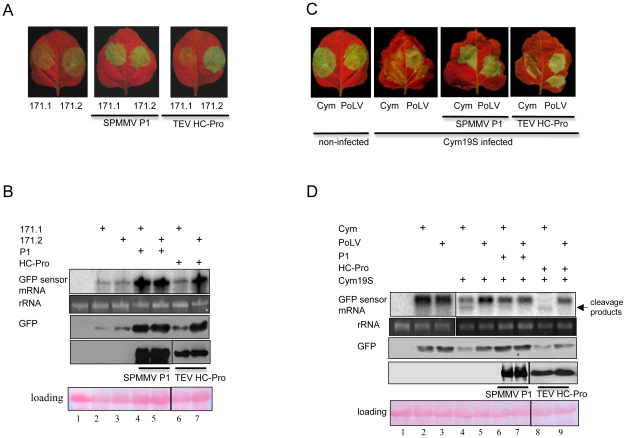
SPMMV P1 inhibits miRNA and viral siRNA loaded RISCs. (A) Constructs expressing tagged SPMMV P1 and TEV HC-Pro proteins were agroinfiltrated with GFP-171.1 and GFP-171.2 sensors. GFP expression was visualized under UV light. (B) RNA gel blot and immunoblot analysis of miRNA sensor RNAs and GFP protein isolated from infiltrated patches. Immunoblot analysis of expression of the different silencing suppressor proteins isolated from infiltrated leaves are also shown. (C) GFP fluorescence of Cym 19S-infected recovering leaves and non-infected leaves infiltrated with GFP-Cym and GFP-PolV sensors and SPMMV P1 and TEV HC-Pro proteins. (D) RNA gel blot and immunoblot analysis of miRNA sensor RNAs and GFP protein isolated from infiltrated patches. Immunoblot analysis of expression of the different silencing suppressor proteins isolated from infiltrated leaves are also shown.

To determine whether SPMMV P1 might inhibit miRNA loaded RISC complexes, we agroinfiltrated GFP171.1 and GFP171.2 sensor constructs [44] with or without the viral suppressors. In these sensors a full complementary miR171 binding site was placed downstream of the STOP codon of GFP ORF allowing miR171-mediated silencing of the GFP171.1 mRNA, while GFP171.2 carried a mutant miR171 target site, which is refractory to miR171-driven RNA silencing [44]. In this experiment the control construct used was TEV HC-Pro. At two days postinfiltration, GFP fluorescence was evaluated under UV light, and then the infiltrated patches were used for RNA and protein isolation. Consistent with previous results, miR171-driven RNA silencing downregulated GFP171.1, but not GFP171.2 at both the RNA and protein level. Strikingly, comparable GFP fluorescence and GFP mRNA and protein were detected in samples infiltrated with GFP171.1+P1 and GFP171.2+P1, indicating that SPMMV P1 efficiently inhibited miR171 loaded active RISC complexes (Figure 3 A, B). As expected for the control, TEV HC-Pro did not inhibit miR171 mediated degradation of GFP171.1 mRNA [22].

Next, we investigated if SPMMV P1 inhibits viral siRNA-loaded active RISC complexes. A previously described system which exploits *N. benthamiana* plants infected with *Cymbidium ringspot virus* (CymRSV) 19 Stop mutant (Cym19S) was used [45]. Cym19S, not expressing the ds siRNA binding silencing suppressor p19, permits a strong RNA silencing response against the virus to be initiated and maintained by enabling viral siRNAs to be loaded into RISC complexes, leading to the recovery of the initially infected plant [45], [46]. At 14–18 dpi of plants carrying Cym19S, the first systemic leaves showed recovery as a consequence of the remarkable amount of active RISCs loaded with siRNAs derived from the virus [22]. Messenger RNAs expressed from the sensor construct GFP-Cym, in which GFP ORF is fused with a ∼200 bp portion of the CymRSV, could be targeted and cleaved by RISC complexes containing Cym19S-derived siRNAs, while GPF-PoLV, in which GFP fused with a ∼200 bp region of *Pothos latent virus*, a virus unrelated to CymRSV, cannot be cleaved, and was used as a negative control [43]. Recovering leaves of Cym19S-infected plants were infiltrated with GFP-Cym and GFP-PoLV alone or with the indicated silencing suppressors. At 2 days post-agroinfiltration (dpa), efficiency of RNA silencing was monitored by visual examination followed by Northern and Western blotting of RNA and protein samples isolated from infiltrated patches (Figure 3C,D).

When the agroinfiltration was performed only with sensors, Northern analysis using a GFP probe detected a shorter hybridizing band, diagnostic for RISC cleavage of the mRNA expressed from the GFP-Cym construct mediated by viral siRNA, while the hybridizing band remained intact in the case of the GFP-PoLV sensor. As expected, the control TEV HC-Pro was not competent to inhibit ss viral siRNA-loaded active RISC complexes, so the GFP-Cym sensor RNA was cleaved [22]. Remarkably, the Northern and Western analyses showed that GFP mRNA and protein levels were similar in GFP-Cym+P1 and in GFP-PoLV+P1 infiltrated samples, and no cleavage product of GFP was detected in the GFP-Cym+P1 infiltrated sample, suggesting that SPMMV P1 efficiently inhibited the slicing activity of the viral siRNA loaded RISC complexes (Figure 3 C,D).

### P1 targets the RISC complex and interacts with AGO1

**Figure 4:**
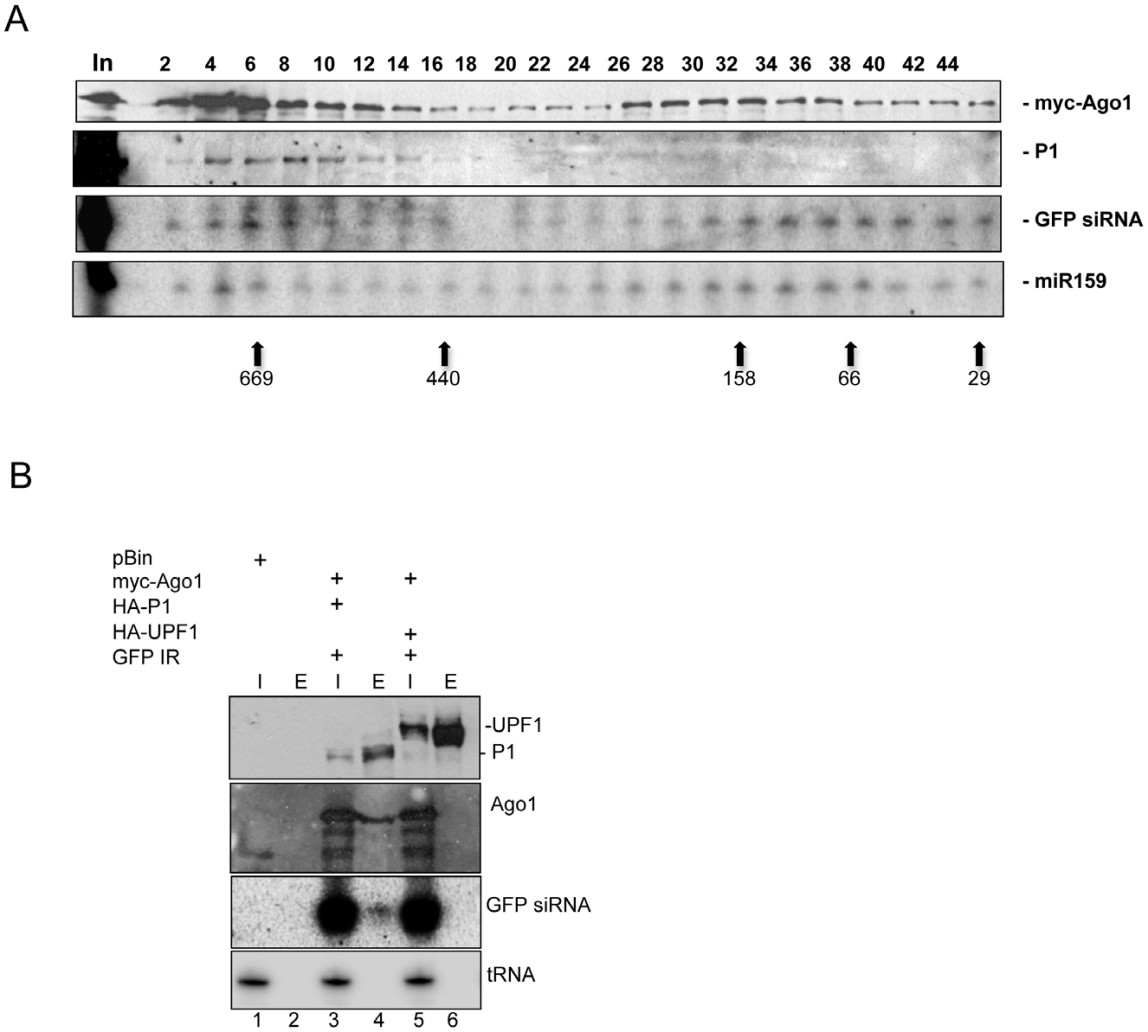
SPMMV P1 cofractionates with AtAGO1 and small RNAs. (A) Western analysis of even fractions of extracts of leaves infiltrated with HA-P1, 6×myc-AtAGO1 and GFP-IR. Anti-myc antibody was used to detect AtAGO1 and anti-HA antibody to detect P1. Northern analysis of RNA isolated from odd fractions. A positive sense GFP RNA probe was used to detect GFP siRNAs processed from GFP-IR. Arrows indicate the elution position of protein molecular weight markers for all panels. The two expected peaks of AtAGO1 correspond to fractions with relatively larger amounts of protein. (B) HA-tagged proteins were immunoprecipitated from extracts of leaves infiltrated with HA-P1+6×myc-AtAGO1+GFP-IR and HA-UPF1+6×myc-AtAGO1+GFP-IR. Myc and HA tagged proteins and GFP siRNAs were detected as in panel (A). tRNA was detected by tRNA specific oligonucleotide probe. Inputs (I) and eluates (E) are indicated above each lane.

The RISC complex is of high molecular weight (>669 kD) in animals [47], [48], contains the catalytic AGO protein, and has intrinsic small-RNA-dependent target cleavage activity. In plants such as *N. benthamiana*, transiently expressed or endogenous AGO1 protein co-fractionates in extracts with small RNAs, and can be found in at least two distinct complexes of above 669 kD and 158 kD [49]. In addition, it was reported that high molecular weight complexes containing viral siRNAs exhibited nuclease activity in vitro and preferentially targeted homologous viral sequences [50]. Having established that P1 inhibits active RISC, we hypothesized that inhibition of RISC requires physical interaction of P1 with AGO1 and small-RNA-containing complexes. To investigate this, we first tested whether P1 co-fractionates with AGO1 and small RNAs on a gel filtration column. N-terminally HA-tagged SPMMV P1 (HA-P1), 6×myc-tagged AGO1 of *Arabidopsis thaliana* (myc-AGO1) [10] and GFP-IR were co-expressed in *N. benthamiana* leaves. At 3 dpi, extracts prepared from infiltrated leaves were fractionated on a Superdex 200HR column. Small RNAs were extracted from each fraction and analyzed by Northern blotting, and the AGO1 and P1 protein contents of fractions were monitored by Western blotting using antibodies raised against the HA and myc tags. Consistent with previous results, GFP siRNAs and miR159 were fractionated in two distinct complexes peaking at >669 and 158 kD, although they appeared in all fractions which also contained AGO1 (Figure 4A). We experienced technical difficulty in separating the protein peaks, and this might reflect the disintegration of the large complexes during chromatography or more likely due to limited availability of the other RISC components. Despite these problems, the infiltrated myc-AGO1 co-fractionated clearly with small RNAs suggesting that GFP siRNAs had been loaded into the myc-AGO1-containing complexes. Interestingly, SPMMV P1 co-fractionated mainly with the 669 kD, but not with the smaller 158 kD myc-AGO1 and small RNA-containing complexes (Figure 4 A).

Next, we investigated if co-fractionation of P1 with myc-AGO1 and small RNAs was due to physical interaction. To test this, we agroinfiltrated HA-P1 with myc-AGO1 and GFP-IR. As a negative control, myc-AGO1 and GFP-IR were agroinfiltrated with HA-UPF1, which is known not to be involved in RISC formation. At 3 dpi, extracts were prepared from infiltrated leaves, and HA-tagged proteins were immunoprecipitated (IP) with an anti-HA antibody. Inputs and eluates of IPs were tested for proteins by Western blotting and for GFP siRNA in Northern blots. The results showed that HA-P1 and HA-UPF1 were expressed at comparable levels and could be efficiently immunoprecipitated from extracts. Importantly, we found that myc-AGO1 coimmunoprecipitated with HA-P1, but not with HA-UPF1, confirming that the interaction between HA-P1 and myc-AGO1 is specific (Figure 4B). Moreover, we found that GFP siRNAs, but not tRNA coimmunoprecipitated exclusively with HA-P1 and myc-AGO1, strongly suggesting that P1 interacts with small RNA-loaded AGO1.

Taken together, we showed that siRNAs derived from GFP-IR became incorporated into myc-AGO1, and that the P1 silencing suppressor specifically interacted with GFP siRNA-loaded myc-AGO1. These results, along with earlier data proving that P1 inhibits si- and miRNA programmed RISC, suggest that the large complex (669 kD) containing AGO1 and small RNAs corresponds to the plant RISC complex.

### The N-terminal 383 amino acids of P1 contains the silencing suppressor domain

**Figure 5:**
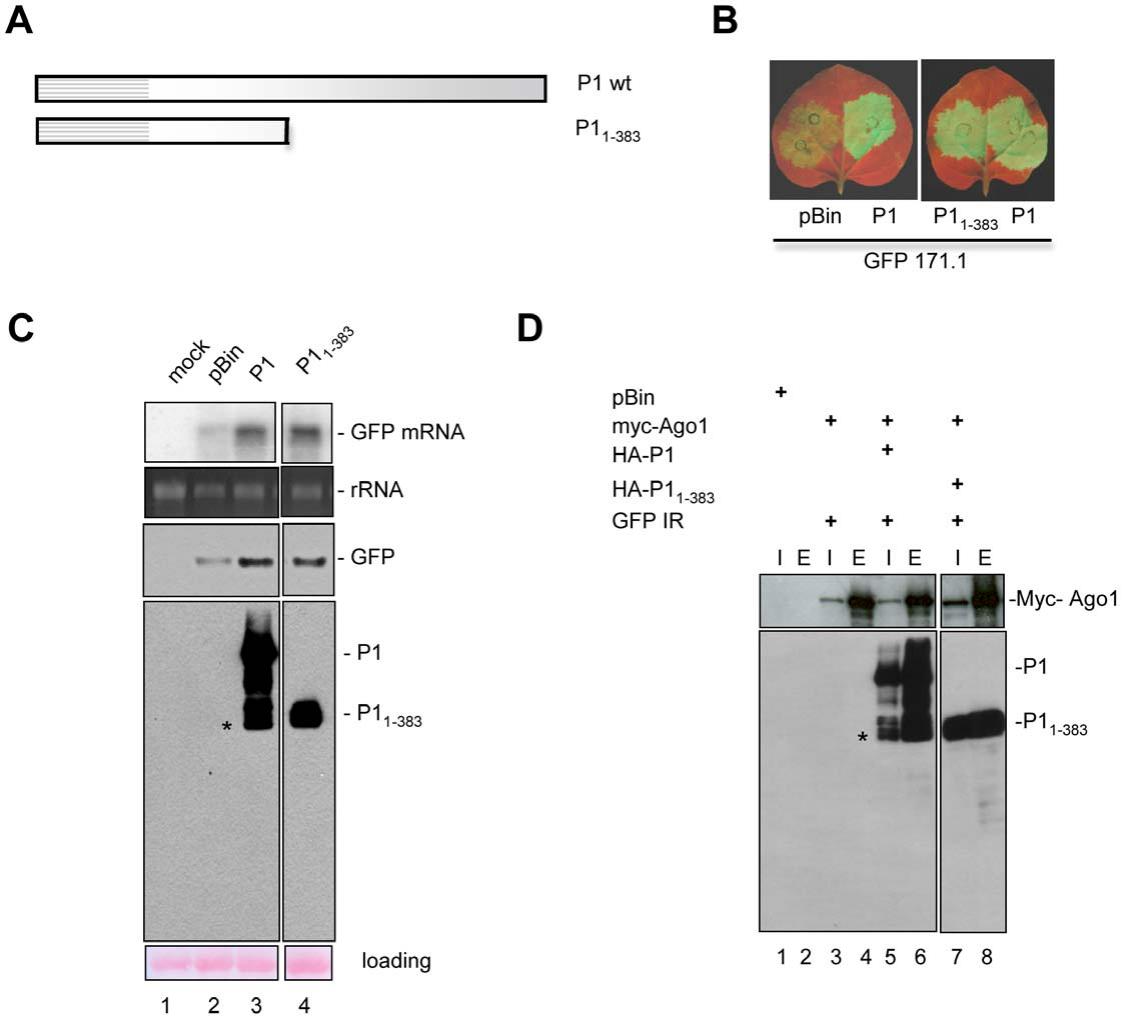
The N-terminal 383 amino acids bears the silencing suppressor domain of SPMMV P1. (A) Graphical representation of P1 deletion mutants. (B) P1 deletion mutants and P1 wt were coinfiltrated with mir171.1. pBIN was used as a negative control. Silencing suppressor activity of the mutants was compared to P1wt under UV light. (C) RNA and protein analysis of infiltrated patches. (D) Interaction of P1 wt and deletion mutants with AtAGO1. 6×myc-AtAGO1 was immunoprecipitated with anti-myc beads. Inputs (I) and eluates (E) were tested for 6×myc-AtAGO1 and for HA-tagged P1 wt and deletion mutants. Stars indicate the non-specific degradation products of full length P1.

P1 contains an extended N-terminal region and a protease domain at its C-terminal end, similar to the P1b of other ipomoviruses [30]; Text S1). To determine the minimal region required for silencing suppression, we constructed P1 mutant truncated from the C-terminal end but retaining the first (N-terminal) 383 aa, designated as HA-P1_1-383_ (Figure 5A). To evaluate its silencing-suppressor activity, the mutant was co-expressed with GFP-171.1 in *N. benthamiana* plants. Visual examination under UV light and analysis of GFP-171.1 sensor RNA and GFP expression showed that HA-P1_1-383_ was an effective silencing suppressor although lacking the entire C-terminal protease domain. Then, we checked the interaction between the deletion mutant of P1 and AtAGO1. To test for interaction, we immunoprecipitated myc-AGO1 from extracts of infiltrated leaves expressing myc-AGO1 and GFP-IR with HA-P1 and HA-P1_1-383_. We used the pBIN61 empty vector as negative control. We found that HA-P1_1-383_ interacted with myc-AGO1 as strongly as wt P1. We concluded that P1 may be composed of two functional domains, the silencing suppressor domain is located at the N-terminal part, and the C-terminal part of P1 contains the protease domain. However, the protease activity of P1 was not analyzed.

### The P1 WG/GW motifs are required for suppressor activity and AGO1 binding

**Figure 6:**
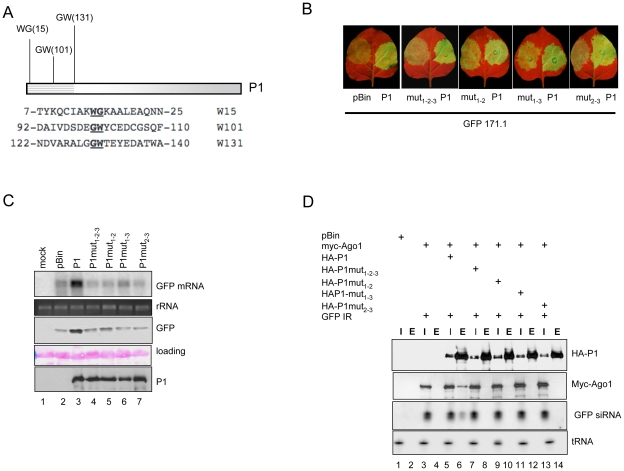
Three WG/GW motifs in the N-terminal part of SPMMV P1 are essential for silencing suppressor activity and AGO1 binding. (A) Graphical representation of the WG/GW signatures in P1. Numbers represent the position of tryptophan residues in P1. (B) P1wt and the double and triple mutants were coinfiltrated with miR171.1 sensor. GFP expression representing silencing suppressor activity of the mutants was compared to that of P1wt. (C) RNA gel blot and immunoblot analysis of infiltrated patches. (D) Interaction of P1wt and point mutants with AtAGO1. HA-tagged P1wt and double and triple mutants were immunoprecipitated with anti-HA beads. Inputs (I) and eluates (E) were tested for 6×myc-AtAGO1, HA-tagged P1wt and double and triple mutants by western blotting. GFP siRNAs were detected by northern blotting.

Our results showed that the N-terminal end of the P1 is required for Ago binding. Further inspection of the N-terminal end of P1 revealed repeating tryptophan-glycine/glycine-tryptophan residues (WG/GW) (Figure 6A and Figure S2), which are identical to the core amino acids of the WG/GW motifs recently found in Argonaute binding proteins, such as Tas3 and RNA Pol V (El-Shami *et al*, 2007; Till *et al*, 2007). Furthermore, analysis of amino acid composition revealed that the regions neighboring the WG/GW residues in P1 are rich in alanine, serine, glutamic acid, asparagine and aspartic acid, providing a context similar to that described for Tas3 and RNA Pol V proteins [35], [51].

This observation prompted us to investigate the significance of the tryptophan residues at the N-terminal end of P1 in silencing suppressor activity. For this, we generated single, double and triple mutants of P1 by replacing tryptophan (W) by alanine (A) residue(s) at positions 15, 101 and 131, individually and in all double (3) and triple (1) combinations, by site-directed mutagenesis. Silencing-suppressor activity of the HA-tagged P1 single mutants was compared to the HA-P1wt, co-infiltrated with the GFP-171.1 sensor construct. Expression analysis of the GFP marker gene reflecting the strength of suppression of RNA silencing showed that suppressor activity of any of the single mutants was not reduced significantly, suggesting that presence of the remaining two tryptophan residues were sufficient to maintain the silencing suppressor activity of P1 (data not shown). In contrast, the suppressor activities of double and triple mutants were greatly reduced. Consistently, wt P1 and the mutants were expressed at comparable level (Figure 6 B,C).

To test whether the WG/GW motifs are required for RNA silencing-suppression because they contribute to AGO1 binding, we tested the interactions between AtAGO1 and P1 double and triple mutants. Myc-AGO1 and GFP-IR were co-infiltrated with double and triple mutants of HA-P1 in line GFP16c/RDR6i *N. benthamiana* plants. As positive control, we used HA-P1wt and as negative control, myc-AGO1 and GFP-IR were infiltrated without P1 wt. At 3 dpi, extracts of infiltrated leaves were used to immunoprecipitate HA-tagged P1 wt and mutant proteins. Western analysis showed that HA-tagged proteins were expressed at comparable levels and were successfully immunoprecipitated. However, probing Western and Northern blots to detect myc-AGO1 protein and GFP siRNA derived from GFP-IR revealed that myc-AGO1 protein and GFP siRNAs were specifically co-immunoprecipitated with P1 wt, but not with any of the double or triple mutants of P1 (Figure 6D).

These results showed that changing at least two out of three tryptophan residues to alanine in the WG/GW motifs of P1 abolished its silencing suppressor activity. Moreover, our analysis showed that the ability of P1 to bind AGO1 depends on the presence of these motifs, suggesting a correlation between AGO1 binding and its activity as silencing suppressor.

### P1 binds endogenous miRNA-loaded AGO1 through direct interaction in vivo

AGO1 of *A. thaliana* is involved both in the miRNA and the antiviral RNA silencing pathways [10], [11], [52]. Our results showed that P1 interacts with AGO1 to inhibit active miRNA and siRNAs loaded RISC.

**Figure 7:**
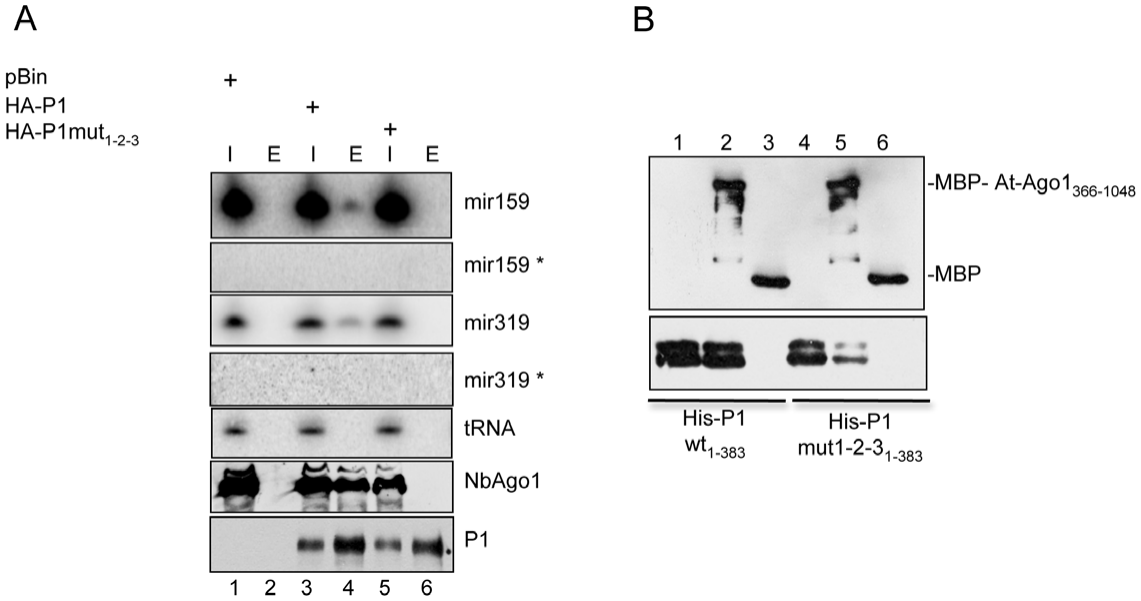
Interaction between P1 and AGO1. (A) P1 interacts with endogenous miRNA loaded Nb-AGO1. Extracts of leaves infiltrated with pBIN, wt HA-P1_1-383_ and HA-P1mut1-2-3_1-383_ were immunoprecipitated with HA-beads. Inputs (I) and eluates (E) were tested for wt P1_1-383_, P1mut_1-2-3_ and for miR319 and mir159. (B) In vitro pull-down assays of MBP-AtAGO1_366-1048_ with wt and triple mutant of 6×His-P1_1-383_. We used 2 mg of MBP-AtAGO1_366-1048_ or MBP to be mixed with 4 mg of 6×His-P1_383_ and 6×His-P1mut1-2-3_1-383_ in a buffer containing 30mM TRIS pH 7.5, 150mM NaCl, 5mM MgCl_2_ and 10% glycerol for 1 hour at 4°C. Then MBP-tagged proteins were isolated on 30 ml maltose resin, washed three times with the same buffer and eluted with 2×SDS loading buffer. Proteins were detected by western blotting using anti-MBP and anti-HIS antibodies.

To get better insight into the mechanism of inhibition of RISC mediated by P1, we performed immunoprecipitations with small RNA-loaded RISCs against P1 (wild type) and P1_mut1-2-3_ (triple mutant)-infiltrated leaf extracts. RNA samples from inputs and immunoprecipitates were probed for presence of two endogenous miRNAs (Figure 7A). The results showed that mature miR159 and miR319 specifically co-immunoprecipitated with wt P1, but not with P1_mut1-2-3_. In addition, we found only mature miRNAs in the eluates of P1 immunoprecipitates, and the star strands for miR159 and mir319 could not be detected in inputs, or in eluates. Thus our results indicated that P1 interacts with endogenous RISC complexes loaded with single-stranded miRNAs (Figure 7A). To test whether P1 interacts directly or indirectly with AGO1 we performed in vitro pull-down assays using recombinant MBP-AGO1_366-1048_ containing the PAZ-MID-PIWI domains and the 6×His-P1_1-383_ N-terminal fragment of wt P1. The results showed that MBP-AGO1_366-1048_ binds wt 6×His-P1_1-383_ efficiently, while the triple mutant P1_1-383_ was bound less strongly by AGO1 protein (Figure 7B). This result strongly suggests a direct interaction between P1 and AGO1 proteins *in vivo* as well.

## Discussion

Available data suggest that virtually all plant viruses encode at least one suppressor and more than 35 individual viral silencing suppressor families have been identified to date. However, the mechanisms of action have been explored only for a few [2], [20]. Among the best-characterized RNA silencing suppressors, the p19 protein of tombusviruses and the HC-Pro protein of potyviruses share the ability to bind and sequester siRNAs, which are the most conserved element of the silencing machinery. SiRNA binding has been postulated as a common and effective strategy to counteract plant defenses against viruses [22]. However, further studies have shown that many viruses use other strategies and can adapt unrelated proteins to target and interfere with different steps in the silencing pathway. For instance, inhibition of siRNA generation by TCV p38 has been described [4], as has the targeting of AGO proteins, the conserved catalytic components of RISC, by viral proteins through protein-protein interactions [10], [25], [53].

In this work we explored the mechanism of silencing suppression mediated by the SPMMV P1 silencing suppressor protein, which interferes with miRNA and siRNA driven RISC activity by binding to the AGO1 subunit of RISC complexes through its WG/GW motifs conserved also in Argonaute binding cellular proteins.

### Characterization of P1 as RNA-silencing suppressor

SPMMV is the only ipomovirus that has a typical potyvirid genome structure with P1 and HC-Pro regions in the C-terminal part of the polyprotein [33]. Other ipomoviruses with available complete sequences do not possess HC-Pro regions [31], [54], [55]. Despite the presence of an HC-Pro in SPMMV, we have found that the role of RNA-silencing suppressor is played by P1.

To better understand the molecular basis of P1-mediated silencing suppression, we analyzed the effect of P1 on different steps of the RNA silencing pathway. We found that P1 does not seem to interfere with the biogenesis of either transgene-derived siRNAs or endogenous miRNAs, since we observed that the accumulation of GFP-IR-derived siRNAs was mostly unaltered in the presence or absence of P1 (Figure 2D). Similarly, accumulation of endogenous miRNAs and their 3′ methylation status were not influenced by the expression of P1, in contrast to the well known TEV HC-Pro suppressor, which binds ds siRNA and miRNA intermediates and partially inhibits their 3′ methylation [22], [41] (Figure 2E). We also showed that P1 failed to bind short and long ds RNAs, in contrast to potyviral HC-Pro and the reovirus sigma3, which efficiently bind ds siRNAs and long dsRNAs, respectively (Figure 2B,C). Thus, this mechanism of silencing suppression seems to be unique among virus-encoded silencing suppressors identified so far.

### P1 inhibits active RISC by interacting with AGO1 loaded with siRNA and miRNA

We tested the effect of P1 on miRNA- and viral siRNA-activated RISC complexes using GFP sensor constructs (Figure 3) and it turned out that P1 efficiently inhibited both types of activated RISC *in vivo*. Moreover, we showed that transiently expressed AGO1 protein was found in a large (>667kD) and an approximately 158 kD protein complexes in size. Interestingly, P1 was found co-fractionating only with the large AGO1 containing complex with GFP siRNAs. This results distinguishes P1 from previously studied silencing suppressors, because it does not inhibit RISC assembly, like small RNA binding suppressors, nor inhibits RISC assembly by promoting degradation of AGO proteins, as it was found in the case of P0 protein of poleoviruses [25], [49], [53]. The 2b protein of CMV FNY strain was also shown to interact with AGO1 *in vivo* and *in vitro* and to inhibit RISC activity *in vitro* [10]. However, it is not known whether 2b prevents RISC assembly or inhibits siRNA loaded RISC by AGO1 binding. In addition, a recent report revealed that 2b proteins of *Tomato aspermy virus* (TAV) and the FNY strain of CMV bind 21nt ds small RNAs [56], [57], so it is not clear, whether 2b protein of cucumoviruses inhibits RNA silencing through siRNA binding, interacting with AGO1 or both. In contrast, P1 binds RISC by interacting the AGO1 subunit of RISC loaded with si- or miRNAs, as shown by our immunoprecipitation studies (Figure 4 and 7). Importantly, P1 interacted with AGO1 containing mature miRNAs but not their star strand; this adds support to our hypothesis that P1 interacts with the AGO1 component of active RISC complexes, and is in line with the efficient inhibition of GFP-sensor silencing by P1. Our cumulative evidence strongly suggests that the P1 interaction with AGO1 is a direct physical interaction. Finally, using mutant P1 proteins with their silencing suppressor activity compromised/abolished, we obtained evidence that silencing suppression and AGO1 binding are linked.

### P1 uses the conserved Ago-hook to bind Argonaute

The WG/GW motifs located at N-terminal part of P1 strongly resemble the evolutionarily conserved GW linear peptide motifs shared by different silencing-related proteins used as “Ago hooks” to interact with Argonaute proteins [35], [58]. Such WG/GW motifs have been described in proteins from different organisms, such as in the largest subunit NRPD1b of the RNA polymerase V in plants [51], the P body-localized human protein GW182 [59], [60], [61], and the Tas3 homologue of the GW182 RITS complex component in yeast [62], [63]. All these proteins can interact with Argonaute proteins [35]. Recently, an RdDM effector KTF1 containing abundant WG/GW motifs and SPT5-like domains has also been identified as an AGO4 binding element [64], [65]. Similarly to cellular WG/GW proteins, our analysis of P1 mutants indicates that the tryptophan residues are essential for interaction with AGO1 and are strictly required for silencing suppressor function (Figure 6).

### Possible models of P1 action

Recent results showed that the AGO-binding domains and the effector domain of GW182 paralogs map in different parts of the proteins [66]. Thus, the modular architecture of the WG/GW proteins that allowed the evolution of Ago-binding elements with positive effects on different RNA silencing pathways, like RNA Pol V, Tas3, GW182 and KTF1 [35], [51], [64], [65], [66], could have been mimicked by a viral protein, although in the case of P1 the effect is negative/suppressive. An attractive possibility to explain the negative effect exerted by P1 on the silencing machinery could be its capacity to outcompete essential AGO1 interacting components of RISC, although in plants these hypothetical AGO1 interactors have not been identified yet. Further experiments will be required to test this possibility and to identify which endogenous elements might be displaced by P1.

We can also postulate alternative explanations for the action of P1. Since small RNA-dependent target cleavage by RISC requires base-pairing between the small RNA and the target RNA, the presence of P1 as an AGO1 interactor might result in covering the small RNA-binding groove of AGO1, thus interfering with base-pairing between the small RNA and the target RNA. This latter possibility is really plausible, because precluding base-pairing between the target RNA and the small RNA would inhibit translation as well, and indeed the importance of translation inhibition in plants has recently been highlighted [12]. In agreement with this, our results with viral siRNA-loaded RISC complexes (Figure 3C and D) show that target cleavage activity did not always correlate with GFP expression (compare lanes 4 and 8 in Figure 3 D); this may indirectly indicate that translational inhibition is hampered by P1 in our system as well.

The efficient binding of AGO1, and inhibition of its function by P1, shown by our experiments suggest that this suppressor might have evolved to bind AGO1 protein with high affinity to inhibit its function. Independently of its final mode of action during suppression, P1 is another example of the extraordinary adaptation of viruses, which are able to target highly conserved key elements of the antiviral silencing response, to be able to complete their infectious cycle. In our study we analyzed only AGO1 as P1 interactor, and we don't know whether P1 is able to interact with other plant AGO proteins. Interestingly, P1 failed to show any effect (Peter Moffett, personal communication) when assayed in an R gene-induced anti-viral response test that is dependent on AGO4-like but not AGO1-like activity [67], suggesting that P1 is not able to target all AGOs.

### Implications of P1-mediated silencing suppression in SPMMV pathogenicity

Our results can also help to explain the pathology of SPMMV, either alone or in synergism with other viruses. Generally speaking, defeat of the host RNA silencing response by a virus equipped with a silencing suppressor requires a high concentration of the suppressor in infected cells, above both the dissociation constant of the suppressor with its target and the intracellular concentration of the target molecule [68]. In SPMMV-infected cells, both existing and *de novo* assembled RISCs, including miRNA- and viral siRNA-loaded RISCs, should be considered as potential targets for P1. At early stages of infection, the concentration of existing active RISC might be much greater than that of the *de novo* viral siRNA-loaded active RISC, and this would lead to the sequestration of P1 mainly by existing active RISC complexes. Consequently, we may hypothesize that the newly formed viral siRNA-loaded RISC could escape from suppression, resulting in low SPMMV titre, mild transient symptoms, and recovery of the plant. However, in cases where plants have additionally been infected with other viruses such as SPCSV, the initial antiviral response might be suppressed by the two-component silencing suppressor system of SPCSV [69], [70] resulting in a much higher titre of SPMMV, which in turn might allow a high concentration of P1 in infected cells. P1 might then efficiently suppress RISCs loaded with both endogenous small RNAs and antiviral RNAs, which would then lead to the synergistic sweet potato disease. High accumulation of SPMMV has indeed been observed in mixed infection with SPCSV, resulting in a severe disease [71]. It is likely that symptom aggravation comes from the fact that both pathogens encode suppressors with complementary effects.

## Materials and Methods

### Virus and plant materials

The African isolate SPMMV-130 was kindly provided by Jari Valkonen (University of Helsinki, Finland) in a sweet potato plant, and maintained in *N. tabacum* Xanthi plants. The complete P1 and HC-Pro regions of the virus were RT-PCR amplified from total nucleic acid extracts using primers 5′CC**TCTAGA** ATGGGGAAATCCAAACTCACTTAC3′ and 5′GT**CCCGGG** TCAATAGAATTGTATCTGTTTAAGTTTACTAG3′ for P1, and 5′CC**TCTAGA** ATGGCAAGTTCTGTTGTACCCAATTTC3′ and 5′GT**CCCGGG** TCAACCAACCTTATAGGTTAACATCTCAC3′ for HC-Pro, and cloned, using the restriction sites highlighted in bold, into competent plasmids for sequencing. The two viral genes were cloned into pBIN-derived constructs for transient expression in *N. benthamiana* leaves. Variants incorporating the tagging element HA were also prepared. Plants were kept in a greenhouse at 22°C under a photoperiod of 12 h/12 h light/dark. Infiltration assays were performed on expanded *N. benthamiana* leaves of plants about 21 days old.

### *A. tumefaciens* infiltration

*N. benthamiana* leaves were infiltrated essentially as previously described [46]. Agrobacterium strains harboring 35S-GFP, GPF-IR, GFP171.1, GFP171.2, GFP-Cym, GFP-PoLV and miR171c precursor were infiltrated with OD_600_ = 0.1. SPMMV 35S-HC-Pro, HA-UPF1 [72], 6×myc-AtAGO1 [10] and RNA-silencing suppressors such as 35S-P1, 35S-P1HC-Pro, HA-P1 were infiltrated with OD_600_ = 0.2–0.3. Infiltrated patches were used for total RNA and protein isolation, then analyzed by Northern and western blotting.

### RNA isolation and hybridization analysis

Total RNA was isolated using TRIZOL reagent. RNA was analyzed on 37% formaldehyde containing agarose gels as described [46]. Small RNAs were analyzed on 12% arcylamide 8M urea gels. RNA isolation from column fractions also was described earlier [73]. Briefly, equal volume of 2×PK buffer was added to each fractions and Proteinase K at final concentration of 80ng/µl. Samples were incubated at 55°C for 15 min. Then, RNA was extracted by phenol-chlorophorm and precipitated with 2,5 volumes of ethanol. After recovering, RNA was resuspended in 50% formamide containing buffer and loaded on 12% arcylamide and 8M urea gels. The gels were blotted and hybridized with riboprobes to detect small RNAs or random primed DNA probes for conventional Northern blots.

### Analysis of the methylation status of small RNAs

Total RNA samples were oxidized, ß-eliminated and detected as described in [41]. Briefly, a total of 10 µg total RNA was dissolved in 17.5 ml borax buffer, pH 8.6, 50 mM boric acid and 2.5 ml 0.2 M sodium periodate was added. The reaction mixture was incubated for 10 min at room temperature in the dark and, after addition of 2µl of glycerol, incubation was repeated. The mixture was lyophilized, dissolved in 50 ml borax buffer, pH 9.5 (33.75 mM borax, 50 mM boric acid, pH adjusted by NaOH) and incubated for 90 min at 45°C. RNA species were then separated on 12% denaturing PAGE blotted and hybridized using ^32^P labeled LNA oligonucleitide probes [74] as described above.

### Immunoprecipitation and immunoblotting

Extracts for immunoprecipitation were prepared in IP buffer containing 30mM TRIS (pH 7.5), 150mM NaCl, 5 mM MgCl_2_, 5mM DTT and 10% glycerol, then incubated for 1 hour at 4°C with beads containing anti-HA (Roche) or anti-myc antibody (Sigma). The beads were then washed with IP buffer. Half of the eluates were used for RNA isolation as described [73]. Commercially available antibodies were used for detecting GFP (Roche), HA-tag (Roche), myc-tag (Sigma), His-tag (Amersham Biosciences) MBP-tag (Sigma). For AGO1 detection we used previously described anti-peptide antibody against *N. benthamiana* AGO1 [49].

### Gel filtration chromatography

Plant extracts for gel filtration were prepared in IP buffer and fractionation was carried out similarly as described earlier [73]. Briefly, 200 µl of extracts were loaded on the Superdex 200HR column and washed with IP buffer with 0.5 ml/min. 25 fractions were collected and after vortexing them for equilibration, each fraction were divided into two for RNA and protein isolation. RNA was isolated, as described above. Proteins were precipitated with 4 volumes of acetone and collected by centrifugation, then solubilized in 2×Laemmli buffer. Proteins were detected by western blotting.

### Site-directed mutagenesis

Site-directed mutagenesis was performed using the Quickchange site-directed mutagenesis kit (Stratagene) according the manufacturer's instructions to generate single, double and triple mutants with oligonucleotides listed in Text S1. Deletion mutants were prepared by PCR using oligonucleotides P1-5′ 5′GGGGATCCCTAGAATGGGGAAATCCAAACTC3′, and P1-383 5′GCGGATCCTCAATCATCAACTTGTGCGTTTAGGGA3′. All mutants were verified by sequencing.

GenBank accession numbers for new viral nucleotide sequence: GQ353374 and for complete SPMMV sequence: NC_003797.

## Supporting Information

Text S1 Alignments of sequences of P1 proteins
(0.07 MB DOC)

Figure S1 Amino acid alignment of ipomoviral P1 proteins, including sequences corresponding to SPMMV P1 (isolate 130), CBSV P1, CVYV P1a+P1b, and SqVYV P1a+P1b. Black background indicates identical residues in at least two sequences, with two shades of gray to account cases where identity exists between two pairs of sequences. Positions of the catalytic triads (H, D/E and S) are highlighted in red lettering and labelled with asterisks, and the cleavage sites are indicated with vertical lines and inverted delta symbols at the end of the sequences for P1 and P1b, while the cleavage between P1a and P1b in CVYV and SqVYV are also shown with similar marks at the corresponding internal sites. WG and GW motifs found in P1 proteins are indicated by orange letterings and yellow boxes. Positions of conserved cysteine residues are indicated with green and blue boxes, respectively for cysteine rich domains near the N-terminus or in a region found to be implicated in the RNA silencing suppression activity of CVYV P1b [32].
(1.92 MB EPS)

Figure S2 Amino acid alignment of the N-terminal region corresponding to one ipomoviral and one potyviral P1 proteins, up to the previously identified region of putative intergeneric recombination [75]. Sequences corresponding to SPMMV isolate 130 P1 and SPFMV P1 are shown. Black background indicates identical residues. The WG and GW motifs found in the N-terminal part of SPMMV P1 are indicated by orange letterings and yellow boxes. Conserved cysteine residues in a cysteine rich domain near the N-termini of the proteins are indicated with green boxes.
(0.80 MB EPS)
