# Supplementary material for: Viral Protein Inhibits RISC Activity by Argonaute Binding through Conserved WG/GW Motifs
Source: PLoS Pathog. 2010 Jul 15;6(7):e1000996. doi: 10.1371/journal.ppat.1000996 (PMC2904775; doi:10.1371/journal.ppat.1000996)
Supplement: Text S1 — Alignments of sequences of P1 proteins (0.07 MB DOC) [file ppat.1000996.s001.doc]

**Supplementary Information for:**

**Viral protein inhibits RISC activity by Argonaute binding through conserved WG/GW motifs**

Ana Giner*^1^, Lóránt Lakatos*†^2^, Meritxell García-Chapa^1^, Juan José López-Moya†^1^, József Burgyán†^2, 3^

^1^Centre for Research in Agricultural Genomics, CRAG, CSIC-IRTA-UAB, Barcelona, Spain

^2^Agricultural Biotechnology Centre, Gödöllő, Hungary

^3^Instituto di Virologia Vegetale, Torino, Italy

* A. G. and L. L. contributed equally to this work

† Corresponding authors: L. L., J. J. L-M. and J. B.

Running title: Viral WG/GW protein inhibits RISC

**Alignments of sequences of P1 proteins**

The sequence of the P1 protein of the SPMMV isolate 130 (GenBank GQ353374) was found similar to the corresponding product of the only other SPMMV isolate [33] with available complete sequence information (GenBank Z73124). The percentage of identity between both SPMMV P1 proteins reached 84 %, although the isolate 130 contained an extra 15 amino acids fragment (sequence IESHSHQNEKLNESL) at position 363 in the published sequence, resulting in a protein with 758 residues, isoelectric point 5.55 and molecular weight 84826.42 instead 743, 5.38 and 83242.91, respectively.

Alignment of the SPMMV P1 protein sequence of isolate 130 with other ipomoviral P1 proteins including CBSV [55] P1 (pI/Mw: 5.66 / 41870.14), CVYV [54] P1a (pI/Mw: 8.79 / 60800.90) and P1b (pI/Mw: 5.19 / 36101.97), and SqVYV [31] P1a (pI/Mw: 8.64 / 61323.74) and P1b (pI/Mw: 5.02 / 36776.84) are shown in Figure S1. While the protease domain at the C-terminal part of P1 and P1b proteins with the position of the corresponding catalytic triads are reasonable conserved for the four ipomoviruses, the region responsible for the internal cleavage of CVYV and SqVYV leading to separation of P1a and P1b products was not conserved in SPMMV nor in CBSV. The sequence of CBSV P1 clearly aligned with CVYV P1b, SqVYV P1b and the C-terminal part of SPMMV P1. The 3 WG/GW motifs found in the N-terminal part of SPMMV P1 were not conserved in the other ipomoviral sequences corresponding to CVYV and SqVYV P1a sequences.

Interestingly, the alignment of SPMMV P1 and CBSV P1 started nearby a putative recombination site predicted earlier [75] between SPMMV and the potyvirus SPFMV [76], a fact that might reflect the divergent origin of the N-terminal potyviral-related extension of SPMMV P1 compared with the ipomoviral-related C-terminal part. This high similarity between the N-terminal part of SPMMV P1 and the corresponding region in the potyvirus SPFMV was proposed to be the consequence of a relatively recent intergeneric recombination event [75]. A comparison between the N-terminal region of SPMMV P1 and the corresponding part of SPFMV P1 (pI/Mw: 9.27 / 73997.41) to the presumed recombination site is presented in Figure S2. In this case, and despite the relatively high conservation (identity 43% in the first 185 residues) that included for instance a cysteine rich domain found in other P1 proteins [75], only the first of the 3 WG/GW motifs found in the N-terminal part of SPMMV P1 was conserved in SPFMV.

**Methods**

Comparison of sequences and alignments

Sequences corresponding to ipomoviral P1 proteins from SPMMV isolate 130, CVYV (GenBank AY578085), SqVYV (GenBank NC010521) and CBSV (GenBank FJ039520) and potyviral P1 protein from SPFMV (GenBank D86371) were aligned with TCoffee using the online facilities at http://www.tcoffee.org/, and displayed in Clustal format using the program Boxshade available at http://mobyle.pasteur.fr/cgi-bin/portal.py, with minor manual refinement. Theoretical isoelectric point (pI) and molecular weight (Mw) were computed for protein sequences with tools available at http://www.expasy.ch/tools/. Percentage of identity between proteins was calculated using blastp algorithm at <http://www.ncbi.nlm.nih.gov/BLAST/Blast.cgi>.

List of oligonucleotides used for site directed mutagenesis

W (15) to A 1 sense 5’TGCATTGCTAAGGCGGGAAAAGCCGCACTTG3’,

W (15) to A 1 antisense 5’CAAGTGCGGCTTTTCCCGCCTTAGCAATGCA3’,

W (101) to A 2 sense 5’ACTCTGATGAGGGAGCGTATTGTGAGGATTGC3’,

W (101) to A 2 antisense 5’GCAATCCTCACAATACGCTCCCTCATCAGAGT3’,

W (131) to A 3 sense 5’GCTCTTGGTGGGGCGACTGAATATGAAGATGC3’,

W (131) to A 3 antisense 5’GCATCTTCATATTCAGTCGCCCCACCAAGAGC3’

Numbers in parenthesis indicate the position of mutated amino acid. Underlined nucleotides show the mutated codons.

**Supplementary Figure legends**

**Figure S1**

Amino acid alignment of ipomoviral P1 proteins, including sequences corresponding to SPMMV P1 (isolate 130), CBSV P1, CVYV P1a+P1b, and SqVYV P1a+P1b. Black background indicates identical residues in at least two sequences, with two shades of gray to account cases where identity exists between two pairs of sequences. Positions of the catalytic triads (H, D/E and S) are highlighted in red lettering and labelled with asterisks, and the cleavage sites are indicated with vertical lines and inverted delta symbols at the end of the sequences for P1 and P1b, while the cleavage between P1a and P1b in CVYV and SqVYV are also shown with similar marks at the corresponding internal sites. WG and GW motifs found in P1 proteins are indicated by orange letterings and yellow boxes. Positions of conserved cysteine residues are indicated with green and blue boxes, respectively for cysteine rich domains near the N-terminus or in a region found to be implicated in the RNA silencing suppression activity of CVYV P1b [32].

**Figure S2**

Amino acid alignment of the N-terminal region corresponding to one ipomoviral and one potyviral P1 proteins, up to the previously identified region of putative intergeneric recombination [75]. Sequences corresponding to SPMMV isolate 130 P1 and SPFMV P1 are shown. Black background indicates identical residues. The WG and GW motifs found in the N-terminal part of SPMMV P1 are indicated by orange letterings and yellow boxes. Conserved cysteine residues in a cysteine rich domain near the N-termini of the proteins are indicated with green boxes.
